# A comparison between equations describing *in vivo* MT: The effects of noise and sequence parameters

**DOI:** 10.1016/j.jmr.2007.12.012

**Published:** 2008-04

**Authors:** Mara Cercignani, Gareth J. Barker

**Affiliations:** a NMR Research Unit, Department of Neuroinflammation, Institute of Neurology, London WC1N 3BG, UK; b King’s College London, Institute of Psychiatry, Department of Clinical Neuroscience, Centre for Neuroimaging Sciences, London SE5 8AF, UK

**Keywords:** MRI, Magnetization transfer, Two-pool model, Relaxation, Human brain

## Abstract

Quantitative models of magnetization transfer (MT) allow the estimation of physical properties of tissue which are thought to reflect myelination, and are therefore likely to be useful for clinical application. Although a model describing a two-pool system under continuous wave-saturation has been available for two decades, generalizing such a model to pulsed MT, and therefore to *in vivo* applications, is not straightforward, and only recently have a range of equations predicting the outcome of pulsed MT experiments been proposed. These solutions of the 2-pool model are based on differing assumptions and involve differing degrees of complexity, so their individual advantages and limitations are not always obvious. This paper is concerned with the comparison of three differing signal equations. After reviewing the theory behind each of them, their accuracy and precision is investigated using numerical simulations under variable experimental conditions such as degree of *T*_1_-weighting of the acquisition sequence and SNR, and the consistency of numerical results is tested using *in vivo* data. We show that while in conditions of minimal *T*_1_-weighting, high SNR, and large duty cycle the solutions of the three equations are consistent, they have a different tolerance to deviations from the basic assumptions behind their development, which should be taken into account when designing a quantitative MT protocol.

## 1 Introduction

The Magnetization Transfer (MT) effect is based on the exchange of magnetization occurring between groups of spins characterized by different molecular environments. In biological tissues, two or more “pools” of protons can be identified: those in free water (the free, or liquid, pool) and those bound to large molecules (referred to as restricted, semisolid, or macromolecular, pool). The latter protons are characterized by a very short transverse relaxation time (*T*_2_) and therefore do not directly contribute to signal intensity in conventional magnetic resonance (MR) images. Nevertheless, it is possible to sensitise an MR experiment to the magnetic resonance characteristics of macromolecular protons by exposing the sample to radio-frequency (RF) energy several kilohertz away from the Larmor frequency. Protons in free water are relatively insensitive to such irradiation, but it can cause saturation of protons in the semisolid pool which, due to their short *T*_2_ and correspondingly large line width, are responsive to irradiation at these frequencies. In these conditions, any exchange of magnetization between pools results in a decreased intensity of the observed MR signal.

From a quantitative MT model based on the exchange between two pools Henkelman et al. [1] derived a signal equation for the continuous wave (CW) case, in which RF irradiation of particular (constant) amplitude and several seconds duration is used to saturate the macromolecular pool. The parameters characterizing the two pools in the model are potentially interesting to measure, and they can be estimated by fitting Henkelman’s equation to a set of MR measurements obtained in the presence of MT pulses with a suitable set of amplitudes *ω*_1_ and offset frequencies Δ*f*.

As CW irradiation is impractical and generally not available for *in vivo* imaging experiments, *in vivo* MT-weighted MRI is generally obtained using the so-called pulsed MT acquisition, in which the long period of saturation is replaced by a much shorter irradiation pulse (typically applied just before each excitation pulse) along with intervals without irradiation (during which data is collected). For data from this type of acquisition, Henkelman’s equation must be modified to allow for the short duration of the saturation pulses relative to *T*_1_ [2]. A number of such modified signal equations for pulsed MT, all based on the same original two-pool model, have been developed [3–5]. While the numerical results published so far suggest reasonable consistency across the solutions predicted by these equations, no direct comparison is available, and the differing conventions and symbols used mean that evaluating discrepancies and similarities between them is not straightforward.

This paper is concerned with the comparison of three of these signal equations—two derived by Sled and Pike [3,6], plus that of Ramani et al. [5]. Ramani et al. used a CW power equivalent approximation (CWPE) [5] where the pulse is simply replaced by a CW irradiation with the mean square amplitude that would give the same power over the interval between MT pulses. By means of the CWPE approximation, Henkelman’s steady state model can be straightforwardly applied to the *in vivo* MRI case, neglecting the imaging elements of the pulse sequence. Due its steady state nature, the implicit assumption within the equation is that the relative signal intensity in data obtained with different MT-weightings only depends on the characteristics of the MT pulse, and that *T*_1_ and *T*_2_ relaxations equally affect all measurements.

As Ramani’s equation does not explicitly model the effects of the excitation pulses and TR, its description of the MT-weighted signal is valid only when the degree of *T*_1_-weighting in the acquisition sequence is minimal. As it effectively assumes that the MT pulse is applied continuously, another parameter likely to affect the accuracy of Ramani’s equation is the duty cycle, i.e. the duration of the MT pulse relative to the repetition period, whose effect has never formally been investigated. Sled and Pike [6] propose an alternative equation which can be fitted directly to the measured signal. Their solution is derived by approximating the pulse sequence as a series of periods of free precession, CW irradiation and instantaneous saturation of the free pool. It has the advantage of incorporating the effect of the excitation RF pulses, and also makes it possible to account for saturation effects of the excitation. Together with this solution, the authors propose also a simpler variant which neglects free precession, thus assuming a succession of instantaneous saturation of the free pool and CW irradiation of the macromolecular pool for the total duration of the interval between pulses. Both equations presented by Sled and Pike for *in vivo* applications require the numerical evaluation of ordinary differential equations (when modeling the rate of saturation of the macromolecular pool with a super-Lorentzian, see next section), at least for the estimation of the effect of the MT pulse on the free pool, and they are therefore computationally intensive. Ramani’s solution has the advantage of being simpler, at the price of its inability to account for the effects of the excitation pulses. A recent paper presented an evaluation of these signal equation, validated using animal data [7]. Here we first review the theory behind them and then use numerical simulations to extend the range of experimental conditions under which each can be tested (investigating how duty cycle, saturation effects of the excitation and noise affect the MT parameters fitted by each of them). We also perform a statistical comparison between MT parameters estimated using each signal equation in healthy brain tissue from *in vivo* data.

## 2 Theory

### 2.1 Coupled Bloch equations

Assuming that the MT effect can be modeled using a liquid pool (*A*) and a macromolecular pool (*B*), the magnetization of either pool can be described by its longitudinal component $\left(M_{z}^{A}\text{,}M_{z}^{B}\right)$ and its transverse components $\left(M_{x}^{A}\text{,}M_{y}^{A}\text{,}M_{x}^{B}\text{,}M_{y}^{B}\right)$. The exchange between pools associated with the transverse components of magnetization can be considered negligible due to the extremely short *T*_2_ associated with the macromolecular pool [2,6]. The coupled Bloch equations for the system can thus be written as follows:

(1) $$\frac{dM_{z}^{A}}{dt}=R_{A}\left(M_{0}^{A}-M_{Z}^{A}\right)-{\mathrm{RM}}_{0}^{B}M_{Z}^{A}+{\mathrm{RM}}_{0}^{A}M_{Z}^{B}+\omega_{1}(t)M_{y}^{A}$$

(2) $$\begin{array}{cc} & \frac{dM_{z}^{B}}{dt}=R_{B}\left(M_{0}^{B}-M_{Z}^{B}\right)-{\mathrm{RM}}_{0}^{A}M_{Z}^{B}\\ & \;\;+{\mathrm{RM}}_{0}^{B}M_{Z}^{A}-(R_{\mathrm{RFB}}(\Delta f\text{,}\omega_{1}(t)))M_{Z}^{B} \end{array}$$

(3) $$\frac{dM_{x}^{A}}{dt}=-\frac{M_{x}^{A}}{T_{2}^{A}}-2\pi \Delta {\mathrm{fM}}_{y}^{A}$$

(4) $$\frac{dM_{y}^{A}}{dt}=-\frac{M_{y}^{A}}{T_{2}^{A}}+2\pi \Delta {\mathrm{fM}}_{x}^{A}-\omega_{1}(t)M_{z}^{A}\text{.}$$

In Eqs. (1)–(4), $T_{2}^{A}$ represents the transverse relaxation time of the liquid pool, $M_{0}^{A}$ and $M_{0}^{B}$ are the fully relaxed values of magnetization associated with the two pools (assumed dimensionless), *R*_*A*_ and *R*_*B*_ are their longitudinal relaxation rates, and *R* is the exchange rate constant. Δ*f* represents the frequency offset of the pulse, while *ω*_1_(*t*) is the time dependent amplitude of the pulse expressed in rad s^−1^ (i.e. the angular frequency of precession induced by the pulse). *R*_*RFB*_(Δ*f*, *ω*_1_(*t*)) is the rate of saturation of longitudinal magnetization in pool *B* due to the irradiation by the amplitude defined by Δ*f* and *ω*_1_(*t*), and depends on the transverse relaxation time of the macromolecular pool, $T_{2}^{B}$. Li et al. [8] show that, in brain tissue, the specra associated with macromolecular pool are better modeled by a super-Lorentzian, (with $\overset{¯}{\omega_{1}^{2}}$ being the average power of the MT pulse), yielding:

(5) $$R_{\mathrm{RFB}}(\Delta f\text{,}\omega_{1})=\overset{¯}{\omega_{1}^{2}}\sqrt{2\pi }\left[T_{2}^{B}\int_{0}^{1}\frac{1}{\left|3u^{2}-1\right|}\exp\left(-2{\left(\frac{2\pi \Delta {\mathrm{fT}}_{2}^{B}}{3u^{2}-1}\right)}^{2}\right)du\right]$$

and we adopt this model here.

We remark here that this notation is not universal. Some authors label the *A* and *B* pools as ‘*F*’ and ‘*R*’, respectively [6], or ‘*f*’ and ‘*m*’ [9,10] and use the symbol *W* instead of *R*_*RFB*_ [6]. The pseudo first-order exchange rates, ${\mathrm{RM}}_{0}^{B}(A\to B)$ and ${\mathrm{RM}}_{0}^{A}(B\to A)$, are often referred to as *k*_f_ (or simply *k*) and *k*_r_.

### 2.2 Sled and Pike’s RP signal equation

Assuming that the pulse sequence consists of an MT pulse followed by an excitation pulse and by a period of recovery, Sled and Pike [6] decompose it into a series of periods where Eqs. (1)–(4) have exact or approximate solutions. These solutions can then be concatenated by imposing the appropriate initial conditions, leading to an expression for the measured signal which is less expensive to compute than numerically integrating the full set of differential equations. The effect of an MT pulse on the macromolecular pool is modeled as a rectangular pulse whose width is equal to the full width at half maximum (*τ*_RP_) of the curve obtained by squaring the instantaneous amplitude of the MT pulse throughout its duration, and whose amplitude is such that the pulses have equivalent average power (rectangular pulse, or RP, approximation). The effect of the pulse on the liquid pool is modeled as an instantaneous fractional saturation of the longitudinal magnetization. Such fractional saturation (*S*_1*A*_) is estimated by solving (numerically) the system of Eqs. (1), (3) and (4) when *R* and *R*_A_ are set to 0.

In matrix form [6], considering the longitudinal components of magnetization only

(6) $$M_{z}(t)=\left[\begin{array}{c} M_{z}^{A}(t)\\ M_{z}^{B}(t) \end{array}\right]\text{.}$$

Instantaneous saturation of the free pool, caused by both MT and excitation pulses, is simply described by multiplying **M**_**z**_ by the matrix **S** (where *θ* is the excitation flip angle)

(7) $$S=\left[\begin{array}{cc} S_{1A}\cos\theta & 0\\ 0 & 1 \end{array}\right]\text{.}$$

The state of the magnetization after a period *t*_1_ (assuming starting time = *t*_0_) is given by the solution to the system of Eqs. (1) and (2) for either free precession [FP] or CW:

(8) $$M_{z}(t_{0}+t_{1})=\exp\{A_{\mathrm{CW}}t_{1}\}M_{z}(t_{0})+[I-\exp\{A_{\mathrm{CW}}t_{1}\}]A_{\mathrm{CW}}^{-1}{\mathrm{BM}}_{0}$$

(9) $$M_{z}(t_{0}+t_{1})=\exp\{A_{\mathrm{FP}}t_{1}\}M_{z}(t_{0})+[I-\exp\{A_{\mathrm{FP}}t_{1}\}]A_{\mathrm{FP}}^{-1}{\mathrm{BM}}_{0}\text{,}$$

with

$$\begin{array}{cc} & A_{\mathrm{CW}}=\left[\begin{array}{cc} -R_{A}-{\mathrm{RM}}_{0}^{B} & {\mathrm{RM}}_{0}^{A}\\ {\mathrm{RM}}_{0}^{B} & -R_{B}-{\mathrm{RM}}_{0}^{A}-R_{\mathrm{RFB}} \end{array}\right]\\ & A_{\mathrm{FP}}=\left[\begin{array}{cc} -R_{A}-{\mathrm{RM}}_{0}^{B} & {\mathrm{RM}}_{0}^{A}\\ {\mathrm{RM}}_{0}^{B} & -R_{B}-{\mathrm{RM}}_{0}^{A} \end{array}\right]\\ & B=\left[\begin{array}{cc} -R_{A} & 0\\ 0 & -R_{B} \end{array}\right]\text{.} \end{array}$$

According to Sled and Pike’s RP approximation, over the time interval *T* between application of MT pulses (typically the time required to excite and collect data for a single *k*-space line of a single image slice), **M**_*z*_ undergoes instantaneous saturation, CW irradiation for a period *τ*_RP_/2, FP for a period (*T* − *τ*_RP_), and CW for another *τ*_RP_/2. After including all thee steps, we can impose the equality

(10) $$M_{z}(T)=M_{z}(0)\text{,}$$

and solve for **M**_*z*_ yielding an equation for the longitudinal components of magnetization. Recalling that the signal observed at readout is

(11) $$\mathrm{SI}(\omega_{1}\text{,}\Delta f)=M_{z}^{A}(\text{TR})S_{1A}\sin\theta \text{,}$$

(where the repetition time TR may be ⩾*T* depending of the details of the image acquisition) it is thus possible to model the MT-weighed signal. The solution to Eq. (10) can be easily computed in matrix form, and we adopt this procedure for all the following experiments.

### 2.3 Sled and Pike’s CW signal equation

A simpler expression is presented in the same paper [6], where the effect of the MT pulse on the macromolecular pool is described by a CW irradiation of duration *T*. In this case, over the same period *T*, **M**_*z*_ undergoes instantaneous saturation, and CW irradiation (of the restricted pool only) for a period *T*. Following the same procedure as described above, a more manageable analytic expression can be derived in this case:

(12) $$\mathrm{SI}(\omega_{1}\text{,}\Delta f)=\frac{(E_{1}-1)(E_{2}-1)(\lambda_{2}-\lambda_{1})S_{1A}M_{z\text{,}\mathrm{CW}}^{A}\sin\theta }{\left(E_{1}-1)(S_{1A}E_{2}\cos\theta -1)(\lambda_{2}-\lambda_{1})+(S_{1A}\cos\theta -1)(E_{2}-E_{1})(\lambda_{2}-R_{A}-{\mathrm{RM}}_{0}^{B}\right)}\text{.}$$

Here $M_{z\text{,}\mathrm{CW}}^{A}$ is the steady state solution obtained by Henkelman et al. [1] for CW irradiation of the restricted pool of duration *T*:

(13) $$M_{z\text{,}\mathrm{CW}}^{A}=\frac{M_{0}\left(R_{A}{\mathrm{RM}}_{0}^{A}+R_{A}R_{B}+R_{B}{\mathrm{RM}}_{0}^{B}+R_{\mathrm{RFB}}R_{A}\right)}{R_{A}{\mathrm{RM}}_{0}^{A}+R_{A}R_{B}+R_{B}{\mathrm{RM}}_{0}^{B}+R_{\mathrm{RFB}}R_{A}+R_{\mathrm{RFB}}{\mathrm{RM}}_{0}^{B}}\text{,}$$

which is equivalent to the first element of the vector $M_{z\text{,}\mathrm{CW}}=A_{\mathrm{CW}}^{-1}{\mathrm{BM}}_{0}$. In Eq. (12),

(14) $$\begin{array}{cc} & \lambda_{1\text{,}2}=\frac{1}{2}\left(R_{A}+{\mathrm{RM}}_{0}^{B}+R_{B}+{\mathrm{RM}}_{0}^{A}+R_{\mathrm{RFB}}\right)\pm \\ & \;\;\frac{1}{2}\sqrt{\begin{array}{c} {\left(R_{A}+{\mathrm{RM}}_{0}^{B}+R_{B}+{\mathrm{RM}}_{0}^{A}+R_{\mathrm{RFB}}\right)}^{2}\\ -4\left(R_{A}R_{B}+{\mathrm{RM}}_{0}^{B}R_{B}+R_{A}R_{\mathrm{RFB}}+R_{A}{\mathrm{RM}}_{0}^{A}+{\mathrm{RM}}_{0}^{B}R_{\mathrm{RFB}}\right) \end{array}}\text{,}\\ & E_{1\text{,}2}=e^{-\lambda_{1\text{,}2}\text{TR}}\text{.} \end{array}$$

We note here that for the specific case of the 3D spoilt gradient echo acquisition described in this paper (see Section 3) *T* = TR.

Sled and Pike also introduce, as a useful index which is believed to correlate with myelin content, the relative size of the macromolecular pool [6] *F*, defined as

(15) $$F=\frac{M_{0}^{B}}{M_{0}^{A}}\text{.}$$

*F* can be fitted directly, by substituting ${\mathrm{RM}}_{0}^{A}=\frac{{\mathrm{RM}}_{0}^{B}}{F}$ in Eq. (13) and previous. More details on these signal equations can be found in [3,6,11].

### 2.4 Ramani’s signal equation

Henkelman’s solution [1] for the CW case is obtained by solving Eqs. (1)–(4) in the steady state, i.e. setting the derivatives on the left hand side to zero. Ramani et al. [5] adopt the same approach in the pulsed MT case, simply replacing the MT pulse with a CW irradiation with the same mean square amplitude

(16) $$\omega_{1\mathrm{CWPE}}=\gamma \sqrt{P_{\mathrm{SAT}}}\text{,}$$

where *P*_*SAT*_ is the mean square saturating field.

In order to ease the comparison between the three signal equations, we break with the terminology of the original paper [5], where the macromolecular fraction *f* (with *f* = *F*/(*F* + 1)) was used, and instead rewrite the Henkelman–Ramani expression using *F* and ${\mathrm{RM}}_{0}^{B}$ to obtain

(17) $$\mathrm{SI}(\omega_{1}\text{,}\Delta f)=\frac{M_{0}\left(R_{B}\left[\frac{{\mathrm{RM}}_{0}^{B}}{R_{A}}\right]+R_{\mathrm{RFB}}(\omega_{1\mathrm{CWPE}}\text{,}\Delta f)+R_{B}+\frac{{\mathrm{RM}}_{0}^{B}}{F}\right)}{\begin{array}{c} \left[\frac{{\mathrm{RM}}_{0}^{B}}{R_{A}}\right](R_{B}+R_{\mathrm{RFB}}(\omega_{1\mathrm{CWPE}}\text{,}\Delta f))\\ +\left(1+{\left[\frac{\omega_{1\mathrm{CWPE}}}{2\pi \Delta f}\right]}^{2}\left[\frac{1}{R_{A}T_{2}^{A}}\right]\right)\left(R_{\mathrm{RFB}}(\omega_{1\mathrm{CWPE}}\text{,}\Delta f)+R_{B}+\frac{{\mathrm{RM}}_{0}^{B}}{F}\right) \end{array}}\text{,}$$

where *M*_0_ is the signal with no MT-weighting (again assuming a constant of proportionality of *c* = 1). Note that while in the equations presented in the previous section, *M*_0_ is the equilibrium magnetization, in Eq. (17) it simply represents the partially recovered magnetization available prior to the application of an MT pulse.

### 2.5 Fitting

All the equations are written in terms of seven parameters: $M_{0}\text{,}R_{A}\text{,}R_{B}\text{,}{\mathrm{RM}}_{0}^{B}\text{,}F\text{,}T_{2}^{A}$ and $T_{2}^{B}$, but these cannot be uniquely determined [1,12]. Constraints are imposed by measuring the observed longitudinal relaxation rate of the sample, *R*_*Aobs*_(=1/*T*_1*obs*_) independently, linked to *R*_*A*_ by Henkelman et al. [1]

(18) $$R_{A}=R_{\mathrm{Aobs}}-\frac{{\mathrm{RM}}_{0}^{B}(R_{B}-R_{\mathrm{Aobs}})}{R_{B}-R_{\mathrm{Aobs}}+\frac{{\mathrm{RM}}_{0}^{B}}{F}}\text{.}$$

A further issue is that dependence of *S*(*ω*_1_, Δ*f*) on *R*_*B*_ is weak, making fitting of this parameter unstable. Since the estimates of the other parameters are largely insensitive to its value, *R*_*B*_ is usually kept fixed at 1 s^−1^ [1,5,6]. This reduces the number of free parameters to 5, which can be estimated by fitting the equation to five or more measurements with different combinations of *ω*_1_(*t*) and Δ*f*.

## 3 Materials and methods

### 3.1 Numerical simulations

In order to compare the three signal equations, and to highlight their shortcomings, we need to test their performance against data corresponding to a known set of parameters. The easiest way to obtain such data is to synthetically produce them, using numerical simulations.

We consider here the case of an MT-weighted spoiled gradient echo acquisition, where off-resonance saturation is achieved using Gaussian pulses (of duration *τ*_*SAT*_) applied once every TR (just prior to RF excitation), while on-resonance excitation is obtained using short 5-lobe sinc pulses (in the presence of a ‘slab selection’ gradient).

Eqs. (1)–(4) can be solved numerically to predict the longitudinal magnetization at the end of the MT pulse (i.e. just before the excitation pulse), $M_{z}^{A}(\tau_{\mathrm{SAT}})$. Since the measured signal intensity is proportional to the transverse magnetization at readout (if we neglect $T_{2}^{*}$ decay):

(19) $$\mathrm{SI}\propto M_{\mathrm{xy}}(\mathrm{readout})=M_{z}(\tau_{\mathrm{SAT}})\sin\theta \text{.}$$

The MT pulse is characterized by its maximum amplitude, *B*_1*SAT*, *MAX*_, by its duration, *τ*_*SAT*_, and by the standard deviation of the Gaussian envelope, *σ*. The excitation pulse is characterized by its maximum amplitude, *B*_1*EXC*, *MAX*_, by its duration, *τ*_*EXC*_, and by its bandwidth, *BW*. Both pulses can be described by their equivalent on-resonance flip angle, given by the integral over pulse duration of *ω*_1_(*t*). Additionally, we assume the excitation pulse to have no effect on pool *B*. The measured signal intensity can be estimated by calculating the solution of Eq. (19) as *M*_*z*_ tends towards a steady state, i.e. after solving Eqs. (1)–(4) for several TRs, until the difference between *M*_*z*_(TR_*n*_ + *τ*_*SAT*_) and *M*_*z*_(TR_*n*+1_ + *τ*_*SAT*_) is less than 0.01% of *M*_*z*_(TR_*n*_ + *τ*_*SAT*_).

We simulate four experiments to probe the effects of different parameters on the fits. Firstly we aim to identify any systematic biases in the three signal equations with respect to variable experimental conditions, by fitting them to noise-free simulated data (Experiments 1–3); next we investigate their robustness in the presence of noise, by using a Monte Carlo approach (Experiment 4). The simulated signal is computed by using a Runge–Kutta ordinary differential equation (ODE) integrator with adaptive step-size control [13]. Spoiling of the transverse magnetization is simulated by setting the transverse components of magnetization equal to zero before the occurrence of every MT pulse. All experiments use the same set of MT parameters $\left(R_{A}\text{,}R_{B}\text{,}T_{2}^{A}\text{,}T_{2}^{B}\text{,}F\text{,}{\mathrm{RM}}_{0}^{B}\right)$, chosen to be similar to values measured previously in white matter [14,15] and shown in Table 1 as ‘test set’. All also use the same excitation pulse parameters (*τ*_*EXC*_ = 3.2 ms, and *BW* = 2.5 kHz), but other values differ:

*Experiment 1:* In the first simulation, we investigate the accuracy of Ramani’s, Sled and Pike’s CW and Sled and Pike’s RP signal equations as a function of duty cycle, by simulating 5 MT experiments using an excitation flip angle of 5°, TR = 30 ms, and varying *τ*_*SAT*_ between 5 and 25 ms, in steps of 5 ms. The full width at half maximum (FWHM) of the pulses varies accordingly between 2.29 (*σ* = 0.97) and 11.45 ms (*σ* = 4.86 ms), in steps of 2.29 ms. Each simulated set consists of 60 points, generated using only two fixed values of *ω*_1*CWPE*_ (250.2 and 850.7 rad s^−1^) (and thus different MT flip angles for each value of *τ*_*SAT*_), following Sled and Pike [6,15], and 30 values of Δ*f* per flip angle. The offset frequency ranges from 400 to 30,000 Hz, sampled at regular interval on a logarithmic scale. The three equations are fitted to the synthetic datasets using the Levenberg–Marquardt method, as implemented in Numerical Recipes [13], to yield the estimated parameters. Numerical derivatives are computed where an analytical expression is unavailable. The test set of parameters (see Table 1) are used to provide the initial parameter estimates, and *R*_*Aobs*_ is obtained by solving Eq. (18) with respect to this quantity. The same ODE integrator used to compute the simulations is used to estimate *S*_1*A*_ at every step for Eqs. (7), (11) and (12).

*Experiment 2:* Secondly, we explore the effects of saturation of the excitation on the estimated parameters, simulating the outcome of six MT experiments using regularly spaced excitation flip angles ranging from 5° to 20° and TR = 30 ms. We fix *τ*_*SAT*_ = 15 ms and keep the other parameters as in the first experiment. With the exception of the excitation flip angle, the input parameters are identical for all six cases.

In order to check that our results are not specific to the choice of the sampling scheme, we repeat Experiments 1 and 2 with an alternative sampling scheme generated using four fixed values of *ω*_1*CWPE*_ (250.2, 450.4, 650.5 and 850.7 rad s^−1^) and 15 values of Δ*f* per flip angle.

*Experiment 3:* As Sled and Pike used two sequences with two different MT pulse durations and two TRs to constrain the estimate of *RM*_0*B*_, we create a synthetic dataset formed by two “sequences” (40 MT points with TR = 30 ms, *τ*_*SAT*_ = 15 ms, *θ* = 5°, 2 MT flip angles equal to 250° and 850°, respectively; and 20 with TR = 45 ms, *τ*_*SAT*_ = 20 ms, *θ* = 6°, 2 MT flip angles equal to 353° and 1202°, respectively). We then compare the accuracy of the MT parameters estimated fitting the three signal equations to such a dataset and to the “single sequence” dataset obtained in Experiments 2 when fixing *τ*_*SAT*_ = 15 ms and *θ* = 5°.

*Experiment 4:* Finally, in order to investigate the sensitivity to noise, we add complex noise with zero mean Gaussian real and imaginary parts to the dataset obtained in Experiment 2 for *τ*_*SAT*_ = 15 ms and *θ* = 5°. We then take the modulus to obtain a noisy data sample. The standard deviation of the Gaussian noise is set to be *M*_0_/Σ, where Σ is the desired SNR in the unweighted image, which we vary over the interval [20, 300]. (The SNR values typically observed in 3D spoiled gradient echo scans from our system, with acquisition parameters similar to those detailed below (see Section 3.2), typically range between 40 and 100 depending on the coil used, resolution, use of parallel imaging, etc.) For each level of noise, we generate 10,000 sets of noisy independent samples and fit the three equations to each set. Look-up tables are used for the super-Lorentzian lineshape, the fractional saturation *S*_1*A*_, and their derivatives, in order to speed up the computation.

### 3.2 *In vivo* data

A single subject (female, 34 years old) was scanned twice on a 1.5 T system (SIGNA Horizon Echospeed, General Electrics, Milwaukee, WI, USA) using a 3D MT-weighted fast spoiled gradient recalled-echo (SPGR) sequence [15] (TR/TE = 28/5.1 ms, Gaussian MT pulses, duration = 14.6 ms, standard deviation = 2.84 ms, bandwidth = 125 Hz, matrix = 256 × 96 × 32, FOV = 240 × 180 × 160 mm^3^, to reconstruct twenty-eight 256 by 256 voxel slices). The excitation flip angle was 5° on the first session and 15° on the second one, the interval between scans was 7 days. On each occasion a dataset of 20 MT points was obtained, using 2 MT pulse flip angles (220° and 820°, corresponding to a CWPE amplitude of 251.1 and 861.2 rad s^−1^), and 10 values of Δ*f* per flip angle. Δ*f* ranged between 400 and 20,000 Hz, and was stepped using a constant logarithmic interval. In addition to the MT data, three 3D SPGRs (TR = 13.1 ms, TE = 4.2 ms, same FOV and resolution as the MT sequence) were also obtained on each occasion, with three different excitation flip angles (*θ* = 25°, 15°, 5°) in order to independently estimate the longitudinal relaxation rate of the system, *R*_*Aobs*_. The body coil was used for signal transmission and the manufacturer’s eight-channel head coil was used for reception. The total scan time was about 45 min.

The study was approved by the Joint Research Ethics Committee of The National Hospital for Neurology and Neurosurgery and the Institute of Neurology, UCL, and the subject gave written informed consent before taking part.

### 3.3 Image analysis

The two datasets (one from each MRI session) were processed on a Unix workstation (Sun Microsystems, Mountain View, CA, USA), as described elsewhere [15]. Briefly: the 20 MT-weighted volumes obtained with the MT-weighted SPGR sequence and the three volumes obtained with the non-MT-weighted SPGR sequence were co-registered to the first MT-weighted volume using a modified [16] version of Automated Image Registration (AIR, available at http://air.bmap.ucla.edu:16080/AIR) [17]. *R*_*Aobs*_ was estimated on a pixel-by-pixel basis by fitting the theoretical SPGR signal equation to the signal in the non-MT-weighted SPGR images, as a function of the flip angle [18]. The 3 MT signal equations were fitted to the remainder of the images (as described for the synthetic data) yielding estimates of the MT parameters. Six bilateral regions of interest (ROIs), three located in white matter (frontal, temporal and internal capsule) and three in gray matter (thalamus, putamen, caudate nucleus) were outlined on the *T*_1_-weighted images obtained from the non-MT-weighted SPGR scan (flip angle = 25°). The 12 ROI outlines were then superimposed on the MT parameter maps, yielding 6 (3 signal equations times 2 flip angles) estimates for each of the following: ${\mathrm{RM}}_{0}^{B}\text{,}F\text{,}T_{2}^{B}$, and $T_{2}^{A}$. Paired sample *T*-tests were used to compare the mean estimated parameters between equation solutions, and two-sample *T*-tests were used to test inequalities between flip angles, considering statistically significant two-tailed *p* values lower than 0.01.

## 4 Results

**Fig. 1:**
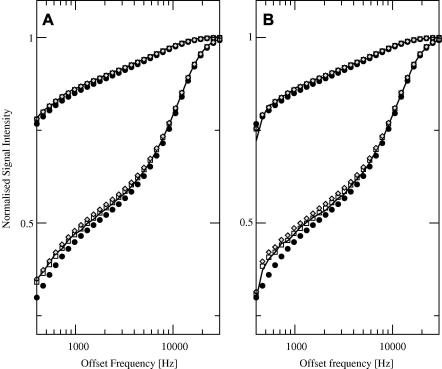
MT spectra simulated using the Bloch equations and the test set in Table 1, together with those simulated using each of the three signal equations (filled circles, Ramani; empty squares, Sled and Pike CW; gray diamonds, Sled and Pike RP) and the same test set, for duty cycle = 50%, flip angle = 5° (A); and duty cycle = 17%, flip angle = 5° (B). All curves are normalized to the maximum intensity.

To provide a qualitative description of the accuracy of the three signal equations, we show in Fig. 1 the MT spectra simulated using Eq. (18) and the test set in Table 1, together with those simulated using each of the three equations and the same test set, for two of the cases explored in Experiment 1 (A: duty cycle = 50%, flip angle = 5°; B: duty cycle = 17%, flip angle = 5°). All curves are normalized to the maximum intensity. As expected, the largest deviations between signal equations are observed at high power, for small offset frequencies, with Ramani’s equation providing the least accurate description. The deviation between Ramani’s predictions and the others are more pronounced for lower duty cycles (Fig. 1B).

### 4.1 Duty cycle effect

**Fig. 2:**
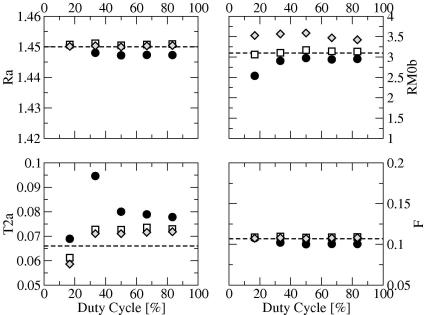
Plot of estimated MT parameters from noise-free simulated data (60 points) against MT duty cycle (in percentage) using Ramani’s (filled circles), Sled and Pike’s CW (empty squares) and Sled and Pike’s RP (gray diamonds) equations. From top to bottom, left to right: *R*_*A*_ [in s^−1^], ${\mathrm{RM}}_{0}^{B}[{\text{in s}}^{-1}]$, $T_{2}^{A}[\text{in s}]$, *F* [unitless]. The dashed line shows the value of parameter used to synthesize the data (test set).

The estimates of $R_{A}\text{,}{\mathrm{RM}}_{0}^{B}\text{,}F$, and $T_{2}^{A}$ against duty cycle are shown in Fig. 2. Results for $T_{2}^{B}$ are omitted as the estimates from all signal equations converged to the test set irrespective of duty cycle. Both of Sled and Pike’s equations are less sensitive to changes in the duty cycle than Ramani’s equation (with the exception of $T_{2}^{A}$), providing very consistent estimates, except for ${\mathrm{RM}}_{0}^{B}$, for which the CW variant yields a value closer to that used to create the simulation. The dependence of Ramani’s on the duty cycle is non-linear, with estimated values tending towards a plateau for duty cycles ⩾50%. For Ramani’s equation, $T_{2}^{A}$ and ${\mathrm{RM}}_{0}^{B}$ are the parameters most sensitive to duty cycle changes. Although more stable, the estimates obtained at larger duty cycles are not necessarily more accurate than those obtained at the lowest duty cycle simulated (17%).

The same experiment repeated with a different sampling scheme yielded almost identical results for duty cycles >17%. For duty cycle = 17%, Sled and Pike’s CW equation converged to $T_{2}^{A}=76.2\;\text{ms}$ and ${\mathrm{RM}}_{0}^{B}=3.31\;{\text{s}}^{-1}$, and the RP variant converged to $T_{2}^{A}=75.0\;\text{ms}$ and ${\mathrm{RM}}_{0}^{B}=3.93\;{\text{s}}^{-1}$, while Ramani’s equation produced results very similar to those obtained with the two-power scheme.

### 4.2 Saturation effect of the excitation

**Fig. 3:**
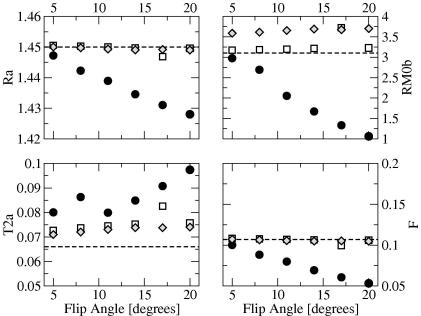
Plot of estimated MT parameters from noise-free simulated data (60 points) against excitation flip angle using Ramani’s (filled circles), Sled and Pike’s CW (empty squares) and Sled and Pike’s RP (gray diamonds) equations. From top to bottom, left to right: *R*_*A*_ [in s^−1^], ${\mathrm{RM}}_{0}^{B}[{\text{in s}}^{-1}]$, $T_{2}^{A}[\text{in s}]$, *F* [unitless]. The dashed line shows the value of parameter used to synthesize the data (test set).

The MT parameter estimates obtained from noise-free simulated data at various flip angles are reported in Fig. 3, again with the exception of $T_{2}^{B}$ which is accurately determined by fitting all signal equations irrespective of the excitation flip angle, and therefore is not shown. As the flip angle (and thus the amount of saturation) increases, the estimates of the MT parameters based on Ramani’s signal equation increasingly deviate from the true values. Additionally, unlike the other parameters, the dependency of the estimated $T_{2}^{A}$ on the amount of saturation does not appear to be monotonic. Sled and Pike’s RP approximation provides the most consistent estimates across flip angles. The CW variant behaves similarly, at least for flip angles lower or equal to 15°, with increasingly biased results at higher flip angles.

We report in Table 1 the estimated MT parameters obtained by fitting the three equations to the simulated noise-free 60 point dataset with excitation flip angle of 5° (minimum saturation effect from the excitation pulse) and duty cycle ≈50% (at which point duty cycle effects have reached a plateau, and the three signal equations seem to provide consistent estimates). All equations give similar results (very close to the ‘test set’ of parameters used in creating the simulation (see Table 1)). The two variants of Sled and Pike’s solution provide more accurate estimates than Ramani’s equation for all parameters, with the CW approximation yielding a more accurate value for ${\mathrm{RM}}_{0}^{B}$, and RP approximation yielding a slightly more accurate value for *F* (which Ramani’s equation tends to underestimate). For all the signal equations, the largest error is in the estimation of $T_{2}^{A}$ (7.6% of true value for Sled and Pike’s RP equation, 10.2% of true value for Sled and Pike’s CW equation, 21.2% of true value for Ramani’s equation). The results of Experiment 3 (fitting the signal equations to a combined dataset obtained with differing “sequences”, i.e. different TR, *τ*_*SAT*_, and flip angle) are also shown in Table 1. The estimates of $T_{2}^{A}$ appear highly sensitive to the combination of acquisition parameters, while all other parameters are not. Interestingly, $T_{2}^{A}$ is underestimated using both of Sled and Pike’s equations when using 2 sequences (and the error becomes larger), while it is generally overestimated when using a single one.

When using the four-power scheme the main difference compared to the two-power scheme was in the estimates of ${\mathrm{RM}}_{0}^{B}$, which were slightly lower for the two variants of Sled and Pike’s solution (between 2.98 and 3.1 for the CW and between 3.3 and 3.4 for the RP approximation).

### 4.3 Sensitivity to noise

**Fig. 4:**
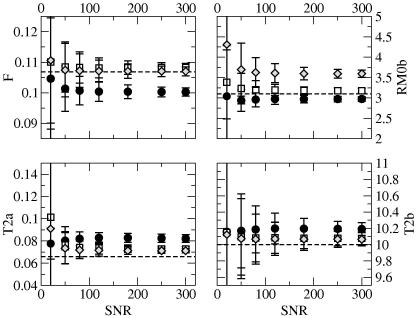
Plot of mean estimated MT parameters from noisy simulated data (60 points) against SNR using Ramani’s (filled circles), Sled and Pike’s CW (empty squares) and Sled and Pike’s RP (gray diamonds) equations. The bars represent the standard deviation. From top to bottom, left to right: *F* [unitless], ${\mathrm{RM}}_{0}^{B}[{\text{in s}}^{-1}]$, $T_{2}^{A}[\text{in s}]$, $T_{2}^{B}[\text{in μs}]$. The dashed line shows the value of parameter used to synthesize the data.

Fig. 4 compares the estimates of the MT parameters using the three signal equations in the presence of noise. Overall, the estimates obtained from the 60 point noisy dataset using any of the three signal equations are characterized by similar precision, although Ramani’s solution seems to provide slightly more robust results than Sled and Pike’s at SNR lower than 120 for ${\mathrm{RM}}_{0}^{B}$ and $T_{2}^{A}$. The two equations proposed by Sled and Pike’s provide very similar estimates (with similar precision at all SNR levels) for $T_{2}^{A}$ and $T_{2}^{B}$. The CW variant provides more accurate values for ${\mathrm{RM}}_{0}^{B}$. The parameter whose estimate deviates most from its true value is (for all signal equations) $T_{2}^{A}$, with Ramani’s estimates deviating more than the others. The standard deviation associated with this parameter, however, is smaller when using Ramani’s equation, at low SNR. The opposite is true (with the two variants of Sled and Pike’s formulation giving very similar performances) at high SNR.

### 4.4 *In vivo* results

**Fig. 5:**
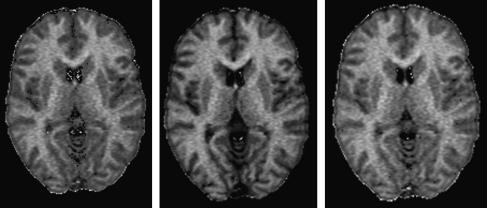
*F* maps obtained fitting Ramani’s (left), Sled and Pike’s CW (middle), and Sled and Pike’s RP (right) equations to the data collected using a flip angle of 5°.

The MT parametric maps obtained by fitting all three equations to the 5° dataset obtained *in vivo* are characterized by similar quality (examples of *F* maps are shown in Fig. 5). Fitting the two equations proposed by Sled and Pike to the 15° dataset was more problematic; in some voxels (most commonly in gray matter, and at the boundary between tissues) the equations gave physically meaningless parameter estimates, particularly for ${\mathrm{RM}}_{0}^{B}$ and $T_{2}^{A}$. It is possible that this is the result of *T*_1_-contrast between tissues and CSF increasing the sensitivity of partial volume effect to motion. Fitting of Eq. (17) appeared to be more robust.

Table 2 shows the values (mean and standard deviation) obtained for each white and gray matter ROI using each signal equation and both flip angles.

#### 4.4.1 5° data (between equations)

Although the estimates of $F\text{,}{\mathrm{RM}}_{0}^{B}$ and $T_{2}^{A}$ obtained with Ramani’s equation were statistically different (*p* < 0.001, providing higher estimates $T_{2}^{A}$ and lower estimates of *F* and ${\mathrm{RM}}_{0}^{B}$) than those obtained with either variant of Sled and Pike’s equations, for all parameters the absolute difference between Ramani’s and Sled and Pike’s estimates was always lower or comparable to the between-voxels standard deviation (within each ROI) (see Table 2). When comparing the two solutions proposed by Sled and Pike, none of the variables differed (*p* values of 0.02 for ${\mathrm{RM}}_{0}^{B}$, 0.6 for *F*, 0.32 for $T_{2}^{B}$ and 0.47 for $T_{2}^{A}$).

#### 4.4.2 15° data (between equations)

Conversely, when comparing the parameters obtained by fitting different equations to the 15° dataset, the mean values were all significantly different (*p* < 0.001), with the exception of $T_{2}^{A}$ obtained using the two variants of Sled and Pike’s solution (*p* = 0.09). The largest differences were in the estimates of *F* and $T_{2}^{A}$ obtained using Ramani’s equation with respect to both Sled and Pike’s equations. The absolute difference between estimates, in this case, were approximately one order of magnitude larger than the between-voxel standard deviation (within each ROI) of the same parameters.

#### 4.4.3 15° data vs. 5° data

The estimates of $T_{2}^{B}$ obtained fitting any of the three equations to the 5° dataset were significantly different from those obtained by fitting the same equation to the 15° dataset (*p* < 0.001). For Ramani’s equation, the estimates of *F* and $T_{2}^{A}$ obtained from the 5° dataset were also significantly different from those obtained from the 15° dataset (*p* < 0.001).

## 5 Discussion

We have shown that the estimates of ${\mathrm{RM}}_{0}^{B}\text{,}F$, and $T_{2}^{B}$ obtained by fitting different equations describing the behavior of the two-pool model under conditions of pulsed MT to proton density-weighted noise-free simulated data with an MT duty cycle of approximately 50% (typical of most *in vivo* applications) are in good agreement and deviate only slightly from the values used to create the simulations. There are some systematic differences, however, with Sled and Pike’s equations typically providing more accurate results. Overall, these findings are in line with those reported by Portnoy and Stanisz [7]. $T_{2}^{A}$ is substantially overestimated when using all signal equations, albeit less so when using the Sled and Pike’s RP approximation. The discrepancy between the estimate of this parameter and the observed *T*_2_ of the system has been observed before [11]. As white matter is known to have multiple free water *T*_2_ components, one of the explanations provided by researchers is that the *T*_2_ measured by spin echo experiments and $T_{2}^{A}$ measured by MT experiments represent different weighted averages of multiple water components. This explanation, however, does not apply to analogous results obtained in gels [1,11]. Similarly, the results of our simulations suggest that such a difference could merely result from the inadequacy of the model to estimate this parameter. It is unsurprising that, for both simulated and *in vivo* data, the largest quantitative difference between the results obtained when fitting different equations to the same (low flip angle) data is in the estimation of this parameter. When the effects of saturation from the excitation pulse are minimized, in fact, the main difference between Ramani’s and Sled and Pike’s equations is in the way the MT pulse effect on the liquid pool is modeled. It is interesting to notice that others have often reported this parameter to be *underestimated* [11], and our own *in vivo* results are consistent with such observations (Table 2). However, when fitting the MT signal equations to simulated data, we observed the opposite trend. A possible explanation for this inconsistency is the sensitivity of Levenberg–Marquardt fitting to the values used as starting points: while for synthetic data we can use the real (test set) numbers, when fitting *in vivo* data we can only provide a ‘best guess’. In order to compensate for this effect we repeated Experiments 1 and 2 running the fitting procedure 10 times, each time perturbing the initial guesses (independently for each parameter) by a random factor from a Gaussian distribution with zero mean and standard deviation equal to 10% of the test parameter [19]. We retained and compared the set of parameters which gave the best fit out of the 10 trials in each case. This did not affect the estimates obtained using Sled and Pike’s equations. $T_{2}^{A}$ obtained using Ramani’s equation was even larger in this case, with all other parameters almost unaffected (data not shown).

An alternative explanation is that we used a larger number of points for simulations than for the human brain data. The range and spacing of the sampling points is likely to affect the results of the fitting [20], and it is interesting to observe that even in the simulated data, $T_{2}^{A}$ is underestimated when using two “sequences” (as recommended by Sled and Pike [6,11]). Contrary to their observations, however, the estimates of ${\mathrm{RM}}_{0}^{B}$ were virtually unaffected by this experimental parameter, and therefore our simulation experiments do not support the need for this type of acquisition. It is not clear why our result in this case should differ from that of Sled and Pike, but it is possible that this is due to our use of a different fitting routine. Because of our simulation results, we chose not to adopt the two-sequence approach for our *in vivo* comparison, as this would have required all the MT-weighted acquisitions to be normalized to the same maximum value, and also might introduce variable degrees of *T*_1_-weightings, which would further violate the assumptions underlying Ramani’s equation.

Although the reason why $T_{2}^{A}$ is so strongly affected by the use of a dual TR protocol is unclear, it has been reported by others that even when using more accurate formulations, the estimates of R and $T_{2}^{A}$ are highly sensitive to the choice of data points [7], and this has been explained as a result of the poor sensitivity of the equations to these parameters, or to systematic errors between the model and the data, which can vary with the sampling points.

The poor sensitivity of the two-pool model to changes in $T_{2}^{A}$ partially explains the robustness of the other parameters against its misestimation. As *T*_2_ can be determined by the use of alternative techniques, the remaining parameters are generally of greater interest. We would like to stress, however, that we are not suggesting the two-pool model is an exact representation of the distribution of macromolecular pools or of their exchange. We simply report that all of these relatively simple signal equations appear to be able to provide consistent and practical information which might be useful in a clinical/clinical research context. Although we did not directly compare the sensitivity of different signal equations to changes similar to those caused by pathology, we have attempted to provide useful guidelines to the choice of the most appropriate one for a particular experimental setting.

Although suggesting optimal sampling schemes is beyond the scope of this paper, it is clear from Fig. 1 that low offset frequencies (<1 kHz) should be avoided when using Ramani’s equation. This is consistent with the set of points used by Ramani et al. in their original paper [5], and with the findings of Portnoy and Stanisz [7], and confirms that in pulsed MT experiments it is difficult to model the behavior of magnetization at low off-resonance frequencies. Our results also confirm the observation of Portnoy and Stanisz [7] that Ramani’s model tends to *underestimate* the signal especially close to the Larmor frequency. This deviation at low frequency offsets is likely to be a consequence of the poor ability of the CWPE approximation to characterize the liquid pool. As noted by Portnoy and Stanisz [7], the cut-off of 1 kHz they empirically determined might vary with field strength and MT pulse amplitude and bandwidth.

We should also note that it is apparent from Figs. 2–4 that *F* and *R*_*A*_ are strongly inversely correlated. This is a trivial consequence of the typical values of *F* in the human brain (of the order of 10^−1^), for which the denominator on the right hand side of Eq. (18) is approximately equal to $\frac{{\mathrm{RM}}_{0}^{B}}{F}$, effectively coupling *R*_*A*_ and *F*. This corresponds to conditions of rapid exchange [9].

The use of numerical simulations allows the investigation of conditions for which it is impractical to acquire *in vivo* data, for example very narrow or very long MT pulses. As expected, and shown by others [7], even in noise-free condition, the estimates of the MT parameters provided by Ramani’s signal equation depend on the duty cycle (in a non-linear fashion), suggesting the need for pulse sequences with a duty cycle of at least 50%. The estimates obtained using Sled and Pike’s equations, on the other hand are, with the exception of $T_{2}^{A}$, less sensitive to this parameter.

We also showed that the degree of *T*_1_-weighting (i.e. of saturation of the excitation) has a large effect on the MT parameters estimated by fitting Ramani’s equation. This is a direct consequence of the assumptions underlying it. We therefore strongly recommend avoiding the use of Eq. (17) to fit data which deviate substantially from those assumptions. The two equations proposed by Sled and Pike, on the other hand, by removing the “steady state” assumption, appear to be less sensitive to this problem. The RP approximation gives estimates that are substantially insensitive to changes in *T*_1_-weighting, while the CW approximation becomes slightly affected by it for excitation flip angles larger than 14° (assuming a TR = 30 ms).

In this respect, the results obtained from synthetic data are in keeping with *in vivo* measurements. Ramani’s equation’s estimates of *F* obtained from the more heavily *T*_1_-weighted data (Table 2, flip angle = 15°) are substantially lower than those obtained with less heavily weighted data, while estimates of $T_{2}^{A}$ are larger. Conversely, when fitting Sled and Pike’s equations to the 15° dataset, the estimates of *F* are slightly larger compared to those from the 5° dataset, although the difference is not statistically significant. A surprising finding from *in vivo* data was that estimates of $T_{2}^{B}$ obtained from all the equations increased slightly with the excitation flip angle, while this was not observed with simulated data. This discrepancy between simulated and real data may be explained by the presence of noise, and by the use of a smaller number of points for the real data. Noise in the raw data critically affects the estimation of MT parameters, as shown by our fourth simulation (Fig. 3). Monte Carlo simulations also suggest that at high SNR (⩾150) Sled and Pike’s estimates (at least of some parameters) are at least as precise as (and generally more precise than) Ramani’s estimates, but that Ramani’s equation provides more precise answers at lower SNR. We also note that at low SNR the uncertainty associated with the estimated parameters is much larger than the systematic difference between the mean parameters estimated by each signal equation. For comparison of the *in vivo* results with simulations, an estimate of the *in vivo* SNR is needed. The typical SNR for our system, measured in white matter on 5°-excitation minimally MT-weighted 3D SPGR images acquired using a 8-channel head coil and the parameters described in the paper, ranges (due to non-uniformity of the receive coil) between 60 and 100 [20]; other systems are likely to be similar. It would be interesting to compare the standard deviations obtained by Monte Carlo iterations to the variance lower bound predicted by Cramer–Rao theory [21], as well as to the standard deviation obtained from real data using the bootstrap method [22].

A further limitation to this analysis of sensitivity to noise is in the use of suboptimal schemes for both synthetic and real data. For all quantitative techniques based on model fitting, the precision and accuracy of the parameter estimates depend on the choice of the sampling points, and we have previously shown [20] that the error in parameter estimates can be reduced by factors around 2 or 3 by using optimized sampling schemes. The precision of the estimates obtained from all three equations could therefore dramatically improve by using a more suitable set of MT points; we recommend such optimization for practical applications, but this was not possible here as we needed identical schemes to allow direct comparison of the three equations. Nevertheless, since the schemes here used were likely to be equally suboptimal for all signal equations (and are similar to those used by the authors of the original papers), we believe that our analysis should not have been unduly “unfair” to any of the solutions tested. It should also be noted, though, that the acquisition protocol reported by Sled and Pike [6,11] is based on a 60-point scheme to sample a single 7-mm thick slice. Here, despite the consequences on both scan time and SNR, we use only 20 points (for *in vivo* experiments) and smaller voxels as, for most clinical applications, such whole brain coverage and thinner slices are likely to be essential. Despite reducing the number of points by two thirds, a scan time of 35 min (required by the protocol we used *in vivo*) is still too long to be feasible in a clinical setting. We also estimated five parameters (${\mathrm{RM}}_{0}^{B}\text{,}F\text{,}T_{2}^{B}\text{,}T_{2}^{A}$ and *M*_0_) directly from the fitting, while the original paper by Sled and Pike [11] recommended the use of an independent estimate of the relative proton density, which allows the MT curves to be normalized to one, reducing to four the number of parameters to be extracted. While this clearly would improve the precision in the estimated quantities, it also lengthens the scan time, making it again less attractive for clinical applications, where the number of acquisitions is typically restricted by time constraints. A better fit could be obtained also by iteratively repeating the fitting using the estimate of *M*_0_ obtained from the current iteration as the starting point for the next. Preliminary results suggest that two iterations should suffice. Furthermore, simulated data suggest that when high SNR and a large number of MT points are available, fitting Sled and Pike’s signal equations provides more accurate results, and therefore is preferable. Between the two variants, the main differences seems to be in sensitivity to the degree of *T*_1_-weighting in the acquisition sequence (as a consequence of saturation from the excitation pulse), suggesting that, in the absence of this confounding factor, the CW variant can be used without major disadvantages, given its reduced complexity. It should be noted, however, that we used a single set of MT parameters to create the simulations, without any attempt to explore the consistency of these results for a different type of tissue (e.g. gray matter). Our results, therefore, are limited to this specific case until confirmed by further experiments.

Regarding the *in vivo* results, it should be noted that we made no attempt to correct *in vivo* data for *B*_1_ inhomogeneities (although the use of the body coil for transmission should provide a fairly uniform *B*_1_ distribution at 1.5 T). Both the measurements of *R*_*Aobs*_, and the MT fitting are affected by deviations from the nominal flip angle [6,18]. However, we expect the error introduced by *B*_1_ inhomogeneity to equally affect the three solutions, and therefore not to have major consequences on the conclusions drawn from our experiments.

In the present work we have restricted our analysis to the comparison of three signal equations derived from the two-pool model to predict signal intensity in pulsed MT experiments, without any attempt to modify them, or compensate for their limitations. Several aspects of MT modeling such as the quantification of the effects of the excitation pulses on the macromolecular pool, which is typically considered negligible [2,6], need to be addressed. It also would be interesting to investigate the dependency of these 3 equations on other sequence parameters such as TR, as this quantity controls the efficiency of magnetization transfer [23]. This analysis would be complementary to the investigation of duty cycle effects we performed, and may confirm whether the relative insensitivity of Sled and Pike’s CW approximation to duty cycle is maintained for all pulse sequences.

Furthermore, it would be interesting to explore possible modifications of Ramani’s equation to account for the imaging parameters of the pulse sequence (for example by incorporating an additional contribution of [1-cos(alpha)]/TR to the CW saturation rate acting on the free pool). Finally, providing reliable information about the optimal number of sampling points and their distribution would yield an additional element towards the choice of the most appropriate equation for a given application. All these areas deserve further investigation, which we hope to pursue in the future.

## 6 Conclusion

We have shown that (1) Sled and Pike’s CW signal equation provides the most accurate estimates of MT parameters in the absence of noise; (2) Ramani’s signal equation is sensitive to changes in the MT duty cycle, although this effect becomes stable for duty cycles ⩾50%; (3) Ramani’s equation is (as expected) not suitable for fitting *T*_1_-weighted data, and doing so leads to underestimates *F* and ${\mathrm{RM}}_{0}^{B}$ when the amount of *T*_1_-weighting is high. An error, albeit of smaller magnitude, is also introduced into Sled and Pike’s CW approximation estimates when the amount of *T*_1_-weighting is high. The RP variant is, on the other hand, extremely robust to this effect; (4) in data with SNR typical of *in vivo* protocols, although the estimates of ${\mathrm{RM}}_{0}^{B}\text{,}F$, and $T_{2}^{B}$ obtained from the fit of all three equations show some differences, the magnitude of the difference is smaller than the between-voxels within-ROI standard deviation, provided that *T*_1_-weighting of the imaging sequence is minimal; (5) Sled and Pike’s equations are slightly less robust than Ramani’s one in conditions of low SNR.

## Figures and Tables

**Table 1:** MT parameter values used to create synthetic data (TEST SET) and results of noise-free simulation with excitation flip angle = 5°, *τ*_*SAT*_ = 15 ms (duty cycle = 50%) using all signal equations

	No. sets	${\mathrm{RM}}_{0}^{B}[{\text{s}}^{-1}]$	*F*	$T_{2}^{B}[\mu \text{s}]$	$T_{2}^{A}[\text{ms}]$	*R*_*A*_ [s^−1^]	*R*_*B*_ [s^−1^] (fixed)
Test set		3.10	0.107	10.0	66.0	1.450	1.0
							
Ramani	1	2.98	0.100	10.0	80.0	1.447	1.0
2	2.98	0.104	10.0	96.1	1.448	1.0
							
Sled and Pike CW	1	3.17	0.108	10.0	72.7	1.451	1.0
2	3.20	0.108	10.0	46.7	1.451	1.0
							
Sled and Pike RP	1	3.59	0.107	10.0	71.0	1.450	1.0
2	3.65	0.107	10.0	45.8	1.450	1.0

The results obtained when using a single set of 60 points (same TR and *τ*_*SAT*_) are also compared obtained when using 2 sets (40 points with the same TR and *τ*_*SAT*_ as the single set, and 20 points with longer TR and *τ*_*SAT*_).

**Table 2:** Mean MT parameter estimates (standard deviation) obtained *in vivo* bilaterally in the six regions listed in the text, using each signal equation, for two flip angles

Flip angle	ROI		Ramani				Sled and Pike CW				Sled and Pike RP			
${\mathrm{RM}}_{0}^{B}$	*F*	$T_{2}^{B}$	$T_{2}^{A}$	${\mathrm{RM}}_{0}^{B}$	*F*	$T_{2}^{B}$	$T_{2}^{A}$	${\mathrm{RM}}_{0}^{B}$	*F*	$T_{2}^{B}$	$T_{2}^{A}$
5	WM1	R	3.73	0.102	9.4	55.8	3.96	0.111	9.1	42.4	5.06	0.108	10.8	42.5
(0.46)	(0.009)	(0.6)	(5.8)	(0.43)	(0.010)	(0.6)	(5.3)	(1.27)	(0.010)	(1.0)	(5.1)
L	4.18	0.103	9.5	49.9	4.16	0.114	9.2	37.6	5.83	0.108	10.8	38.2
(0.71)	(0.009)	(0.5)	(6.3)	(0.40)	(0.008)	(0.5)	(4.2)	(1.49)	(0.008)	(0.8)	(4.3)
WM2	R	2.91	0.100	10.3	56.6	3.15	0.109	10.0	43.2	3.60	0.106	10.6	42.9
(0.29)	(0.009)	(0.7)	(6.9)	(0.32)	(0.009)	(0.7)	(6.4)	(0.48)	(0.009)	(0.9)	(6.2)
L	3.10	0.087	10.6	59.7	3.42	0.094	10.2	46.4	4.22	0.093	9.3	45.9
(0.62)	(0.010)	(0.9)	(5.5)	(0.70)	(0.010)	(0.9)	(6.0)	(1.42)	(0.011)	(1.1)	(5.9)
WM3	R	2.75	0.108	10.6	53.0	3.03	0.115	10.2	40.4	3.29	0.115	11.5	39.6
(0.40)	(0.014)	(0.6)	(7.0)	(0.39)	(0.013)	(0.5)	(6.3)	(0.60)	(0.015)	(1.4)	(6.1)
L	3.35	0.094	11.1	57.8	3.65	0.103	10.8	44.6	4.45	0.100	10.0	44.3
(0.43)	(0.008)	(0.8)	(5.5)	(0.49)	(0.009)	(0.8)	(5.6)	(0.93)	(0.008)	(0.8)	(5.4)
GM1	R	1.89	0.080	9.6	59.1	2.34	0.083	9.1	46.3	2.31	0.087	8.7	44.6
(0.24)	(0.013)	(0.7)	(6.4)	(0.18)	(0.013)	(0.7)	(7.4)	(0.34)	(0.014)	(1.4)	(6.7)
L	2.57	0.079	9.6	55.6	2.90	0.086	9.2	41.6	3.42	0.085	8.5	41.3
(0.46)	(0.007)	(0.9)	(4.5)	(0.47)	(0.008)	(0.9)	(3.8)	(0.92)	(0.007)	(0.7)	(3.5)
GM2	R	1.67	0.057	8.6	65.5	2.12	0.062	8.3	52.7	2.25	0.062	6.3	51.9
(0.23)	(0.005)	(0.5)	(2.4)	(0.50)	(0.007)	(0.4)	(4.8)	(0.54)	(0.006)	(0.6)	(4.4)
L	1.86	0.056	8.2	64.7	2.33	0.060	7.9	52.5	2.79	0.060	6.0	51.8
(0.37)	(0.005)	(0.5)	(2.2)	(0.50)	(0.007)	(0.5)	(4.5)	(1.12)	(0.006)	(0.6)	(4.2)
GM3	R	2.04	0.056	8.4	65.5	2.79	0.060	8.0	55.1	2.99	0.061	6.1	53.7
(0.50)	(0.011)	(0.6)	(5.2)	(0.53)	(0.015)	(0.6)	(9.3)	(0.96)	(0.012)	(1.2)	(7.7)
L	2.05	0.059	8.0	62.9	2.43	0.065	7.7	49.8	5.06	0.064	6.5	49.3
(0.44)	(0.011)	(0.6)	(5.6)	(0.42)	(0.012)	(0.5)	(9.5)	(1.27)	(0.011)	(1.1)	(8.2)
														
15	WM1	R	2.53	0.068	12.6	78.0	3.10	0.138	12.1	33.2	3.43	0.135	12.1	33.0
(0.38)	(0.004)	(1.1)	(4.7)	(0.32)	(0.009)	(1.1)	(1.7)	(0.57)	(0.009)	(1.1)	(1.6)
L	3.07	0.063	12.2	78.7	3.43	0.129	11.7	33.1	3.98	0.125	11.7	33.0
(0.51)	(0.005)	(0.9)	(8.3)	(0.37)	(0.010)	(0.8)	(3.0)	(0.59)	(0.009)	(0.9)	(3.0)
WM2	R	2.50	0.063	12.4	86.2	3.20	0.130	11.9	34.9	3.62	0.127	11.7	34.7
(0.45)	(0.009)	(0.6)	(15.5)	(0.27)	(0.015)	(0.6)	(4.9)	(0.37)	(0.015)	(0.6)	(4.8)
L	2.64	0.063	13.4	86.8	2.86	0.129	12.9	35.5	3.16	0.126	12.8	35.2
(0.40)	(0.009)	(0.9)	(12.1)	(0.33)	(0.016)	(0.8)	(3.6)	(0.44)	(0.015)	(0.8)	(3.6)
WM3	R	2.64	0.061	13.2	89.0	2.97	0.128	12.7	35.7	3.32	0.124	12.7	35.5
(0.45)	(0.007)	(0.9)	(7.8)	(0.45)	(0.012)	(0.8)	(2.4)	(0.57)	(0.012)	(0.8)	(2.3)
L	2.34	0.068	14.0	100.2	3.03	0.143	13.6	40.8	3.30	0.140	13.6	40.1
(0.40)	(0.007)	(0.6)	(16.6)	(0.34)	(0.012)	(0.5)	(6.7)	(0.48)	(0.012)	(0.5)	(6.5)
GM1	R	2.98	0.038	10.8	114.5	2.31	0.092	10.3	39.5	2.80	0.087	10.2	39.7
(0.39)	(0.002)	(0.7)	(12.2)	(0.29)	(0.004)	(0.7)	(3.2)	(0.58)	(0.005)	(0.7)	(3.2)
L	2.83	0.042	12.1	112.1	2.43	0.099	11.6	39.4	2.80	0.095	11.5	39.4
(0.46)	(0.003)	(0.4)	(10.2)	(0.31)	(0.006)	(0.4)	(3.2)	(0.46)	(0.005)	(0.4)	(3.1)
GM2	R	1.95	0.036	12.7	122.9	1.44	0.087	11.9	41.9	1.57	0.084	11.9	41.9
(0.26)	(0.004)	(0.7)	(10.8)	(0.12)	(0.008)	(0.6)	(2.7)	(0.12)	(0.007)	(0.6)	(2.7)
L	1.91	0.032	14.9	145.2	1.30	0.078	14.1	49.6	1.41	0.076	13.4	4.0
(0.27	(0.003)	(1.2)	(12.7)	(0.09)	(0.006)	(1.1)	(4.2)	(0.20)	(0.006)	(1.12)	(3.3)
GM3	R	2.11	0.027	11.4	160.0	1.32	0.072	11.2	47.1	1.50	0.069	11.2	49.8
(0.59)	(0.007)	(2.3)	(20.1)	(0.14)	(0.009)	(0.9)	(5.1)	(0.21)	(0.009)	(1.0)	(4.3)
L	2.02	0.036	13.1	142.7	1. 53	0.086	12.6	47.1	1.70	0.084	12.5	47.0
(0.57)	(0.005)	(1.6)	(22.3)	(0.50)	(0.008)	(1.5)	(3.2)	(0.64)	(0.008)	(1.5)	(5.0)

WM1, frontal; WM2, temporal; WM3, internal capsule; GM1, thalamus; GM2, putamen; GM3, caudate. ${\mathrm{RM}}_{0}^{B}$ is measured in s^−1^; *F* is unit less; $T_{2}^{B}$ is measured in μs; $T_{2}^{A}$ is measure in ms.
